# Social and Physical Distance/Distancing: A Corpus-Based Analysis of Recent Changes in Usage

**DOI:** 10.1007/s41701-021-00107-2

**Published:** 2021-06-25

**Authors:** Christopher S. Butler, Anne-Marie Simon-Vandenbergen

**Affiliations:** 1 School of Music, Humanities and Media, University of Huddersfield, Huddersfield, UK; 2 Department of Linguistics, Ghent University, Ghent, Belgium

**Keywords:** Covid-19, Social distance/distancing, Physical distance/distancing, Semantic change

## Abstract

Since the outbreak of the Covid-19 pandemic and its dramatic spread in the early months of 2020, the term *social distancing* has rapidly become a key term in public and private discourse. At the same time, *social distance, physical distance* and *physical distancing* have become current in the same context. This paper examines these terms in (samples of) four corpora of British English (BNC, ukWaC, NOW 2019 and NOW 2020), with the following aims: (i) to study the frequency and usage of these phrases in corpora of different kinds, representing texts created both before and during the Covid-19 pandemic; (ii) to determine whether the recent spread of the phrases in the Covid-19 context has entailed any shifts in the collocational profile of the constituent words. By looking at the most frequent collocations over time we establish to what extent the corpora reflect stability and change in patterning and to what extent the external factor of the pandemic outbreak has far reaching consequences for the lexical semantics of the language. The case of *social distance/distancing* has special relevance to accounts of semantic change through the sudden and radical shifts in the collocational profile of the items concerned.

## Background and Aims

The spread of terms related to Covid-19, and in particular the term *social distancing*, has been widely reported (see “Previous Literature on Covid-19 Related Language” Section below). In itself the appearance of a new term as a result of particular social events is a normal phenomenon, and so is the spread in such cases of (technical) terms from one domain into another or into everyday language. However, a number of factors make *social distancing* particularly interesting from a semantic point of view. First, it is not a technical term which has found its way into everyday language through external factors (in contrast with such terminology as *the R number,* see “Previous Literature on Covid-19 Related Language” Section), nor is it a case of metaphorical transfer from a source domain for the ideological and persuasive representation of a donor domain (in contrast with metaphors triggered by catastrophic events such as wars, financial crises or even epidemics, as studied by e.g. Chiang & Duann, 2007; Chifane, 2013; Lakoff, 1991), and nor is it the well-known phenomenon whereby existing words are used in new senses as a result of new inventions or other socio-cultural factors (such as *screen* for a wooden partition and then more frequently for a television or computer screen), and where the old and new senses live together (Hollmann, 2009: 531). *Social distance/distancing* are not single words but phrases, and the collocation of the two component parts in the new Covid-19 context makes their place in and effect on the lexicon more complicated. We shall investigate this by looking not only at the phrases themselves, their usage and meaning, but also at the collocational behaviour of their component words *social*, *distance* and *distancing*. The aim is to look for patterns and shifts in the most frequent collocations.

Secondly, that the terms *social distance* and *social distancing* are felt to be new in the senses Covid-19 generated is clear from reactions that have been voiced by individuals as well as by such official bodies as the World Health Organization (see e.g. Gale, 2020) and the European Centre for Disease Prevention and Control (ECDC, 2020). The argument is that while the population should observe physical distance measures, social ties and connectedness should be maintained. Hence, the term *social distancing* has been argued to be potentially misleading and *physical distancing* has been recommended in its place. For that reason we have included *physical distance/distancing* in our study. This observation of unease regarding the meaning of the term raises the question of what sense speakers of British English tend to give to the terms *social distance/distancing* if not the ‘new’ one. Speakers understand terms through contextual factors and distributional information plays an important part in interpreting a term (Mc Donald & Ramscar, 2001). For that reason the collocational behaviour of the items concerned in different corpora would hypothetically tell us which contextual clues most typically call up the different senses of the key terms.

Thirdly, that different groups in society may readily interpret terms in different ways is evident. For example, Robert (2008: 80-81) mentions not only prototypes and discourse situations but also “personal attractors” as “meaning attractors”, i.e. “elements which attract/steer a term’s interpretation in a particular direction”. The example is *instrumental*, which a linguist out of context will interpret differently from a musician (Robert, 2008: 81). In the case of *social distance/distancing* there is one interpretation which has very suddenly invaded the whole of society and ousted other interpretations, at least without a discourse context which would steer interpretation towards another sense. Even though the Covid-19 related sense can be shown to have existed in the medical domain before (cf. Medical Dictionary, 2009), its sudden and radical take-over means that the more usual sense of an attitude (according to the OED, cf. “Previous Literature on Covid-19 Related Language” Section below) has had to make way for the spatial sense. Such a movement from more subjective to more objective goes against the usual semantic regularities in language change (Hopper & Traugott, 1993), and is also for that reason worth investigating.

The above considerations have led to the hypothesis that we are dealing with a new term which deeply affects the semantics of the lexical fields concerned. In order to test this hypothesis, this paper examines the terms *social distance/distancing* and *physical distance/distancing* in a set of corpora, with the following aims: (1) to study the frequency and usage of our key terms in corpora of different kinds, representing texts created both pre- and post-Covid-19; (2) to examine the meanings of the terms in these corpora; (3) to determine whether the recent spread of the terms in the Covid-19 context has entailed any shifts in the semantic fields in which the words belong. The usage of the terms is investigated by looking at their most frequent collocations, on the basis of which the degree of similarity or difference between the corpora with respect to these terms is established, and new developments detected.

## Previous Literature on Covid-19 Related Language

It is not surprising that the most disastrous global health crisis since the so-called ‘Spanish flu’ pandemic of 1918 has created a large volume of academic discussion. However, we have found rather few published accounts of work on the detail of lexis and grammar in texts centred on Covid-19. There is work on macro-level aspects of language in relation to the pandemic, such as Kelly’s (2020) interesting paper on ‘Languages and the coronavirus crisis’, which collates information from newspapers and other printed media which is of interest to language professionals and concentrates on matters of discourse and communication which may have an impact on language policies. Tan et al. (2020) is a set of short articles, under the overall heading of ‘Covid-19 insights and linguistic methods’, which adopt a range of methodologies, both qualitative and quantitative and at both macro- and micro-levels, for the study of documents concerned with Covid-19. Here there is some analysis of corpus materials in relation to a number of terms which have become frequent in discussions of the pandemic, but these do not include those relating to social or physical distance/distancing.

Scholars working on the Oxford English Dictionary, however, have published a set of very useful reports of corpus analyses undertaken to detect items for consideration as additions to the dictionary. Oxford English Dictionary (2020a), a blog from 9 April 2020, discusses the influence of epidemic diseases on vocabulary over the centuries, and comments as follows: “Social distancing, first used in 1957, was originally an attitude rather than a physical term, referring to an aloofness or deliberate attempt to distance oneself from others socially—now we all understand it as keeping a physical distance between ourselves and others to avoid infection.” In Oxford English Dictionary (2020b), a blog from 15 April 2020, they summarise recent trends, as revealed by analysis of the Oxford monitor corpus of over 8 billion words of web news content from 2017 onwards, updated every month. They show that the frequencies of words denoting the virus and the disease increased dramatically from December 2019 to March 2020. They also isolate statistically significant collocates of the word *coronavirus*, presenting tables of the top 20 collocates in January, February and March 2020. In addition, they compute the collocations which were statistically more frequent in these three months as compared with their frequency in the corpus as a whole. The word *distancing* was at position 3 of the keywords list for March, but absent from the lists for January and February. The frequency of *social distance/distancing* leapt from a very low figure to 190 per million words in March 2020. In Oxford English Dictionary (2020c), a blog from 15 July 2020, the keywords for April to June 2020 are given: *distancing* is at positions 8, 6 and 17 respectively in those months, showing a decline from the figures for the earlier months, other aspects of the pandemic having become dominant in the lists. Oxford English Dictionary (2020d) notes that “specialist scientific and medical language is increasingly prominent in everyday discourse” and gives information on, for example, various abbreviations for Covid-19, drugs which have proved useful in treating coronavirus sufferers, the R number, ventilators, field hospitals and community transmission/spread. Finally, Oxford Languages (2021), a report on lexical changes in English during 2020, has a section on Covid-19 which includes a subsection on ‘*Social distancing*, *lockdown*, and other measures’.

A blog from April 2020 on the website of the Department of English Language and Linguistics at the University of Birmingham (Hunston, 2020) also comments briefly on changes to the English lexicon in the wake of the Covid-19 pandemic, including the huge upsurge in the use of *social distancing* and to a lesser extent *social distance*.

## Data

We have used a range of corpora of different types in our study, all taken from British English usage: the XML version of the British National Corpus (BNC) (Burnard, 2007); the ukWaC50 corpus, which is a random 50% sample of the material in the UK component (ukWaC) of the WaCky Wide Web project (see Baroni et al., 2009); and that part of the NOW (News on the Web) corpus (see Davies, 2016-) drawn from the .uk internet domain.

The BNC, with just over 112 million words, is composed of text samples, usually no longer than 45,000 words, around 90% dating from 1985 to 1993, not restricted to particular subjects, registers or genres and containing 90% written and 10% spoken language. The written component covers imaginative (fictional/literary/creative) and informative (natural, pure, applied and social sciences, world affairs, commerce and finance, arts, belief and thought, leisure) areas. The spoken component contains material, mainly conversational, sampled according to age, gender and social group, and also context-governed material from educational, business, public/institutional and leisure domains.

The ukWaC corpus, compiled in 2007 and containing around 2 billion word tokens, is the UK component of the WaCky Wide Web project (for an introduction to the project as a whole and to ukWaC see Ferraresi et al. 2008; for more detail on ukWaC see Baroni et al. 2009). The aim was to build a corpus which would be comparable, in terms of the spread of documents, to previous ‘balanced’ corpora such as the BNC. The compilers made a detailed comparison with the BNC, by means of wordlists of nouns, verbs and adjectives, concluding that ukWaC contains most of the vocabulary present in the BNC, also that ukWaC contains a higher proportion of words concerned with the Web, education and ‘public sphere issues’, while the BNC has more words characteristic of narrative fiction, spoken language and texts concerned with political and economic issues. A random 50% sample of this corpus (ukWaC50) is available through the corpus analysis facilities at Lancaster University.

The NOW (News on the Web) corpus (Davies, 2016-) is a daily augmented corpus which differs sharply from the BNC and ukWaC in that it is restricted to just one genre, that of web-based newspaper and magazine articles from a wide range of country domains, with data from 2010 onwards. As of 11 November 2020 it contained more than 11.3 billion words.

The availability of these corpora facilitates two types of analysis which are relevant to our aims. Firstly, the overall similarity of the BNC and ukWaC in terms of their make-up offers the possibility of establishing a set of patterns of usage for each of our key items which is stable across almost two decades and can be taken as a baseline for the properties of these items in ‘general British English’, i.e. across a range of types of text. Secondly, by analyzing sections of the GB component of the NOW corpus which are limited by date, we can study the usage of our target items in web-based newspaper and magazine articles in the months before the Covid-19 pandemic took hold and during recent months in which there has been intense discussion of the virus situation. The periods we have chosen are July to December 2019 (henceforth referred to as ‘NOW-E’ (early), with ca. 89 million words) and January to June 2020 (‘NOW-L’, (late) ca. 153 million words).

## Software Tools for Corpus Analysis

BNC and ukWaC50 were analysed by means of the CQPWeb interface to the Corpus Workbench software, provided together with the corpora available through UCREL at Lancaster University (see Hardie, 2012). The NOW corpus was analysed using the software provided together with Mark Davies’s collection of corpora at Brigham Young University.

## Data Analysis

### Frequencies of Key Items in the Corpora

Table 1 shows the frequencies of our target items in the corpora under investigation. It should be noted that all frequencies refer to word forms rather than lemmas, so for instance *distance* covers both nominal and verbal uses of the form *distance* but does not include *distances*, *distancing* or *distanced*. The frequencies per million words (henceforth pmw), highlighted in bold type, are the most relevant here.

**Table 1:** Frequencies of key items in the four corpora

Item	BNC (ca. 112 M words)		ukWaC50 (ca. 1.12 B words)		NOW-E (ca. 89 M words)		NOW-L (ca. 153 M words)	
	Freq.	Per million words	Freq.	Per million words	Freq.	Per million words	Freq.	Per million words
Distance	6595	**58.83**	81,371	**72.20**	4194	**47.19**	8468	**55.27**
Distancing	138	**1.23**	566	**0.50**	72	**0.80**	20,762	**135.50**
Social	41,649	**371.53**	378,955	**336.23**	27,113	**305.09**	62,607	**408.61**
Social distance	38	**0.34**	105	**0.09**	1	**0.01**	676	**4.41**
Social distancing	0	**0**	2	**0**	0	**0**	19,088	**124.58**
Socially	1499	**13.37**	11,519	**10.22**	723	**8.14**	2275	**14.85**
Socially distance	0	**0**	0	**0**	0	**0**	336	**2.19**
Socially distanced	0	**0**	2	**0**	0	**0**	542	**3.54**
Socially distances	0	**0**	0	**0**	0	**0**	8	**0.05**
Socially distancing	0	**0**	1	**0**	0	**0**	119	**0.78**
Physical	9435	**84.16**	110,600	**98.13**	6076	**68.37**	10,965	**71.56**
Physical distance	11	**0.10**	105	**0.09**	5	**0.06**	92	**0.60**
Physical distancing	0	**0**	1	**0**	0	**0**	751	**4.90**
Physically	1938	**17.29**	15,600	**13.84**	1,857	**20.90**	3,118	**20.35**
Physically distance	0	**0**	1	**0**	0	**0**	16	**0.10**
Physically distanced	1	**0**	1	**0**	0	**0**	22	**0.14**
Physically distances	0	**0**	0	**0**	0	**0**	0	**0**
Physically distancing	0	**0**	0	**0**	0	**0**	9	**0.06**

The following conclusions can be drawn:

- The frequencies of the forms *distance* across the four main bodies of text under scrutiny are of the same order of magnitude, though with some variation (range 47.19–72.20 pmw).
- S*ocial* is much more frequent than *distance*, but again the frequencies across corpora are quite similar (range 305.09–408.61 pmw).
- *Distancing*, on the other hand, is rare in BNC, ukWaC50 and NOW-E, but over 100 times more frequent in NOW-L.
- The frequency of *social distance* is very low in BNC, ukWaC50 and NOW-E. It is higher in NOW-L, but not greatly so.
- *Social distancing* is entirely absent from BNC and NOW-E and virtually so from ukWaC50, but is very frequent indeed in NOW-L.
- *Socially* has similar, and fairly low, frequencies in all four corpora (range 8.14–14.85 pmw).
- Combinations of *socially* with a form of verbal *distance* are absent from BNC and NOW-E, and virtually ukWaC50 too, but are present, albeit at low frequency, in NOW-L.
- *Physical* has similar frequencies across the corpora (range 68.37–98.13 pmw).
- *Physical distance* is very infrequent in all four bodies of text.
- *Physical distancing* is not found (apart from one occurrence in ukWaC50) in any body of text apart from NOW-L, where it is 25 times less frequent (pmw) than *social distancing*.
- *Physically* has similar frequencies across the four corpora, slightly higher than those of *socially* (range 13.84–20.90 pmw).
- Combinations of *physically* with a form of verbal *distance* are absent from BNC and NOW-E, and virtually so from ukWaC50, but are present, though still with very low frequency, in NOW-L.

It is clear, then, not only that there is a surge in references to matters of distancing in the 2020 texts (NOW-L), but also that by far the most common way of referring to these matters is by the use of expressions using *social* rather than *physical* and that *distancing* is used much more frequently than *distance* in these contexts, *social distancing* being by far the most frequent choice. These findings clearly corroborate, and also supplement, those reported in Oxford English Dictionary (2020a, b, c, d) and Oxford Languages (2021).

In what follows, we will first explore in some detail the patterning of *distance*, *distancing* and the adjectives *social* and *physical* in the corpora, using a combination of concordances and collocational information, in order to understand better any semantic shifts which have occurred in recent usage and the relationship between *social* and *physical* descriptors of distancing.

### Distance

#### Frequencies

We begin our exploration of the ways in which distance is characterised by examining the words which occur most frequently immediately before the word form *distance* in the corpora.^1^ Here and elsewhere we will concentrate on the 40 most frequent lexical collocates at this position^2^. This procedure will not capture every modification of the head noun, since there are also coordinated instances such as (1) and (2) where there are modifiers at other positions to the left of the head noun as well as immediately before it:

1. The greater size of many firms, and the **social and spatial distance** between owners and workers or top managers and lower managers … (BNC BNF 1059)
2. … how do you get them to work within **walking, cycling or tram-ride distance** of work? (BNC AB6 1097)

Such examples are infrequent compared with those with a single modifier, so any additional collocates are very unlikely to affect the top 40 list. Specific coordinated instances will, however, be considered separately in Section “Both Physical and Social Distancing” because they can provide useful information on what properties of the concept of distance are being regarded as distinct (see, for example, *social* and *spatial* in (1)).

**Fig. 1:**
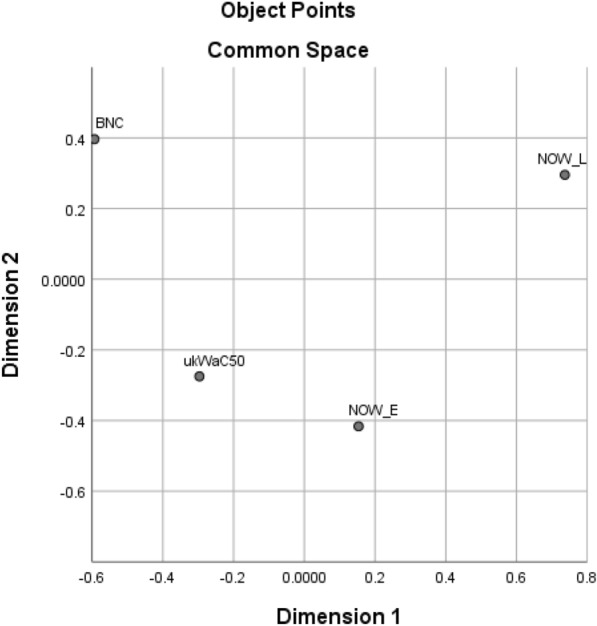
Multidimensional scaling plot of distances between corpora on the basis of numbers of shared collocates in top 40 for *distance*

Table 2 shows all the lexical collocates found in the top 40 for any of the four corpora and which top 40 list(s) they occur in, and Table 3 indicates how many collocates are shared by the top 40 of each pair of corpora, while Fig. 1 presents a multidimensional scaling plot prepared using SPSS version 26, with the matrix of numbers of shared collocates as input data.^3^

**Table 2:** Presence of lexical collocates immediately before *distance* in top 40 for each corpus

Collocate	BNC	ukWaC50	NOW-E	NOW-L	Collocate	BNC	ukWaC50	NOW-E	NOW-L
Certain	×	×	×	×	Travel			×	×
Considerable	×	×	×	×	Respectful	×			
Good	×	×	×	×	Route	×			
Greater	×	×	×	×	Fixed	×			
Long	×	×	×	×	Thinking	×			
Middle	×	×	×	×	Critical	×			
Reasonable	×	×	×	×	Right	×			
Safe	×	×	×	×	Equal	×			
Same	×	×	×	×	Discreet	×			
Short	×	×	×	×	Correct	×			
Striking	×	×	×	×	Viewing	×			
Total	×	×	×	×	Geographical	×			
Touching	×	×	×	×	Increasing	×			
Walking	×	×	×	×	Large	×			
Commuting		×	×	×	Race		×		
Fair		×	×	×	Easy		×		
Minimum		×	×	×	Focusing		×		
Spitting	×		×	×	Star		×		
Driving	×	×		×	Shortest		×		
Physical	×	×		×	Online		×		
Social	×	×		×	Mph			×	
Full	×	×	×		Ironic			×	
Great	×	×	×		Passing			×	
Small	×	×	×		Decent			×	
Stopping	×	×	×		Hearing			×	
Travelling	×	×	×		Socially				×
Far	×	×			Two-metre				×
Little	×	×			Metre				×
Working	×	×			2m				×
Vertical	×		×		Metres				×
Average		×	×		Keep				×
Extra		x	×		Maintain				×
Longer		×	×		Appropriate				×
Maximum		×	×		One-metre				×
Shorter		×	×		Maintaining				×
Similar		×	×		Emotional				×
Draw			×	×	Physically				×
Horizontal			×	×	Recommended				×
Marathon			×	×	Keeping				×
Significant			×	×

**Table 3:** Numbers of collocates of *distance* shared by top 40 lists for each pair of corpora

	ukWaC50	NOW-E	NOW-L
BNC	25	21	18
ukWaC50		28	20
NOW-E			23

It can be seen from Table 3 that each corpus shares between 45% (BNC/NOW-L) and 70% (ukWaC50/NOW-E) of its top 40 collocates with each other corpus, though BNC and ukWaC50 share more collocates with each other and with NOW-E than with NOW-L. This suggests two conclusions:

- The relatively high number of common collocates provides evidence for a set of items immediately before *distance* which constitute a common collocational usage which is stable across both time (BNC < ukWaC50 < NOW) and genre (BNC/ukWac50 ‘general written British English’)/NOW ‘newspapers and magazines on the web’), so going beyond the similarity of just BNC and ukWaC50 referred to earlier.
- The fact that BNC and ukWaC50 share fewer collocates with NOW-L than with NOW-E suggests that there may be new items in NOW-L which have downgraded some of the less frequent ‘common usage’ collocates.

Let us examine each of these possibilities in a little more detail.

**Fig. 2:**
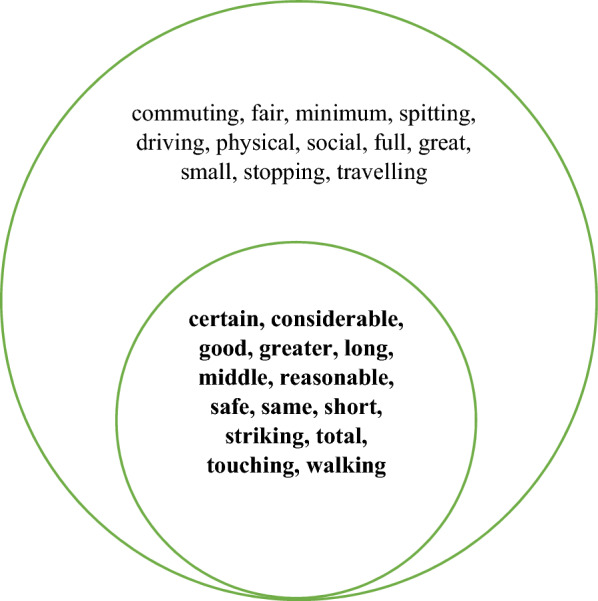
The collocates present in the top 40 for *distance* in all four corpora (bold) and those present in three corpora

Table 2 shows that 14 collocates are present in the top 40 for all four corpora. If we add in the collocates that are present for just three corpora, there are an additional 12. These collocates are shown in Fig. 2.

**Fig. 3:**
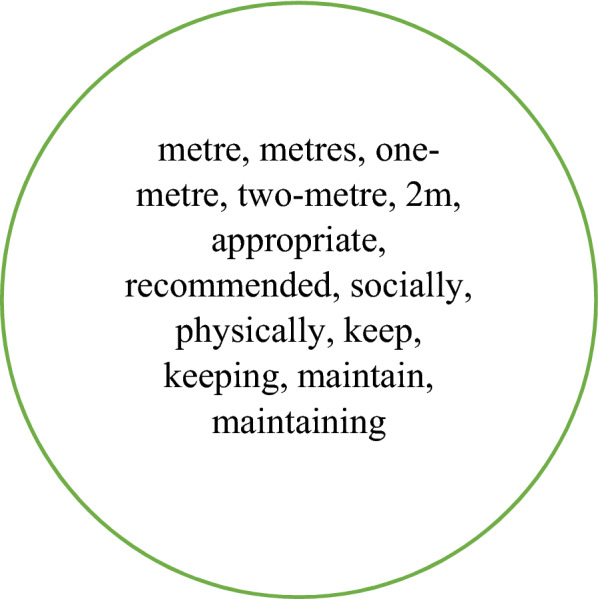
Collocates of *distance* which are unique to the top 40 in NOW-L

Table 2 also indicates that 14 collocates are unique to NOW-L. These are shown in Fig. 3. It is noteworthy that many of them are likely to be concerned with distance away from something or someone. This, together with the presence of *maintain(ing)*, *keep(ing)*, *socially*, *physically*, strongly suggests a connection with social/physical distancing. This conclusion is also, though more weakly, supported by the inclusion of *recommended* and *appropriate*. With the adverbs *socially* and *physically* we are clearly dealing with the verbal rather than the nominal form of *distance*.

#### Semantic Analysis

**Fig. 4:**
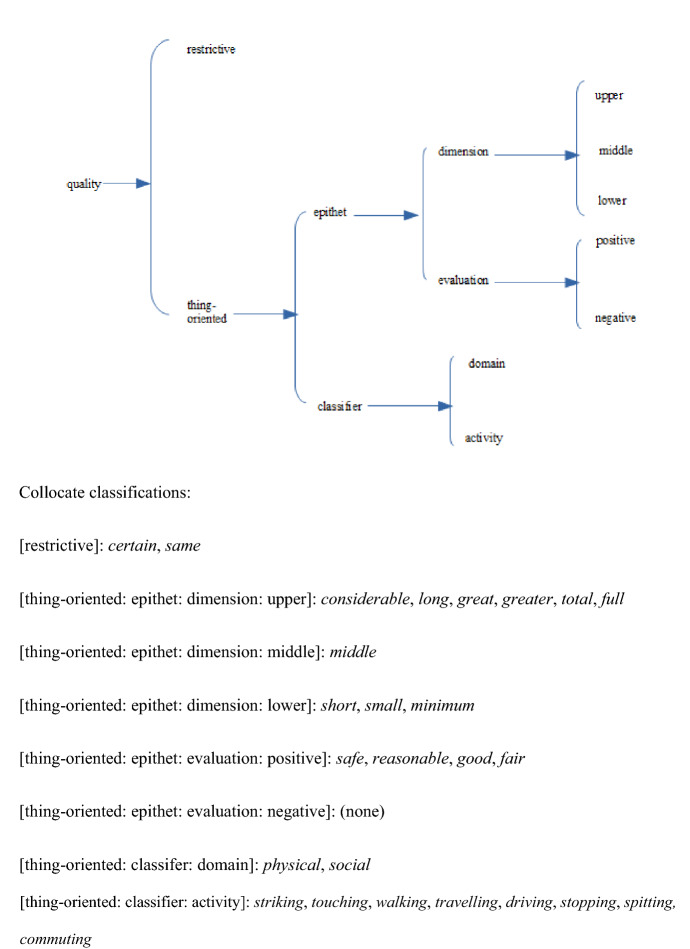
Semantic network for quality type for the 26 collocates of *distance* shared by three or all four corpora

The collocates can be classified into a number of semantic sub-classes. Classification of the 26 collocates shared by three or all four corpora yields a picture of the general usage of *distance* in general British English over a period of some three decades. A similar classification of the collocates which characterise NOW-L provides a picture of the major changes in the collocational patterning of *distance*.^4^ Our semantic analysis of adjectival modifiers is based on relevant selections from the semantic distinctions made for ‘Quality senses’ in the networks presented in Tucker (1998: §7.3). The network in Figure 4 classifies the ‘general usage’ collocates.

The classification needs some clarification. First, the collocates *reasonable*, *good* and *fair* are classified as expressing positive evaluation, which is in these cases an evaluation of the dimension ‘length’ of the distance. The items have lost their judgemental meaning in favour of a dimensional sense of ‘moderate to long’. *Safe* is the only truly evaluative epithet, which nevertheless also implies a position on the length scale: distance which is judged contextually great enough. Second, the classifiers shared by all four corpora refer to an activity which can be carried out within the distance from a point of reference. In the case of *striking*, *touching*, *spitting* and to a lesser extent *walking,* the activity can necessarily only be carried out if the distance is small. The collocates thus express a low value on the dimension ‘length’. *Stopping* and *commuting* on the other hand require a longer distance, and hence express a higher value on the dimension scale. The activity classifiers *travelling* and *driving* express neutral values on the dimension scale. The domain classifiers *physical* and *social* are dealt with in Section “Social and Physical Distance/Distancing” below.

**Fig. 5:**
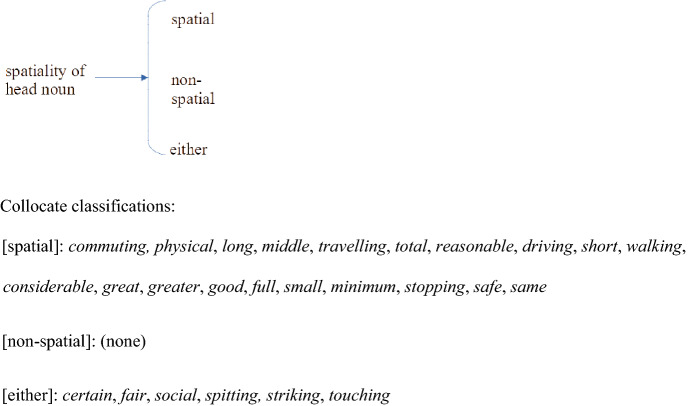
Semantic network for spatiality of quality for the 26 collocates of *distance* shared by three or all four corpora

Cutting across this semantic classification of the collocates is the distinction between the spatial or literal sense of *distance* and its metaphorical or non-spatial sense. With respect to the 26 collocates occurring in the top 40 of three or all four corpora there are in this respect three possibilities: collocates which occur with spatial *distance* only, collocates which occur with non-spatial *distance* only, and those which occur with either sense. The 26 shared collocations have the distribution shown in Fig. 5.^5^

Not only are there only six types, out of our list of 26, which occur before non-spatial distance, but the frequency of the tokens in this context is also negligible. The only exception is *social distance.* See “Social and Physical Distance/Distancing” Section.

The spatial and non-spatial uses of *striking* are illustrated in BNC examples (3) and (4) respectively:

(3) Let us assume that a cat stalks a duck until it gets within **striking distance**. The cat then breaks cover and makes a final uncovered dash along the length of the jetty.(BNC B74)
(4) If Mr Major, having presided over an economic recovery, should also win the next election, the Tories would come within **striking distance** of Sir Robert Walpole’s record of 21 unbroken years in office, set between 1721 and 1742. (BNC AK9)

It will be noted that example (4) shows a metaphorical use of the whole expression rather than a non-spatial use of *distance* only. This applies to *fair*, *spitting*, *striking* and *touching distance*.

This usage is different from that of *certain* in its non-spatial context, where it is *distance* itself which is non-spatial, while the meaning of *certain* remains the same. Compare BNC examples (5) and (6) for spatial and non-spatial uses of *distance* respectively:

(5) Our safety regulations dictate that they be a **certain distance** from the crowd, so that if anything did go wrong, the aircraft would be able to er get out of the way (BNC KRT)
(6) The aims of their travels were peaceful and this sets them at least a **certain distance** apart from the crusaders. (BNC BMV)

In our four corpora there are collocates which occur with non-spatial *distance* only by definition, as they are classifiers specifying the non-spatial senses in which *distance* should be understood. Examples are: *aesthetic, chronological, emotional, cultural, evolutionary, genetic, historical, judgemental, moral, phylogenetic, political, psychological.* Their frequencies are low and they do not make it to the top 40.

We turn now to the difference between NOW-E and NOW-L, as these allow us to make clear comparisons between pre-Covid and Covid periods, within the same genre. The six most frequent collocates in NOW-E–*long*, *short*, *walking*, *touching*, *safe*, *striking*–occur in all four corpora. All but one of these come lower down the NOW-L list, by an average of four ranks. The exception is *safe*, which has clear implications of a context in which the danger of close proximity is foregrounded. The six most frequent collocates in NOW-L include *long*, *short* and *walking*, as well as *safe*, but at positions one and three we find *social* and *socially* respectively. The top three–*social*, *safe*, *socially*–account for 46.25% of the total frequency of all 40 collocates. We saw in Section “Frequencies” (Fig. 3) that the collocates which are unique to NOW-L also strongly reflect the connection with Covid-19.

**Fig. 6:**
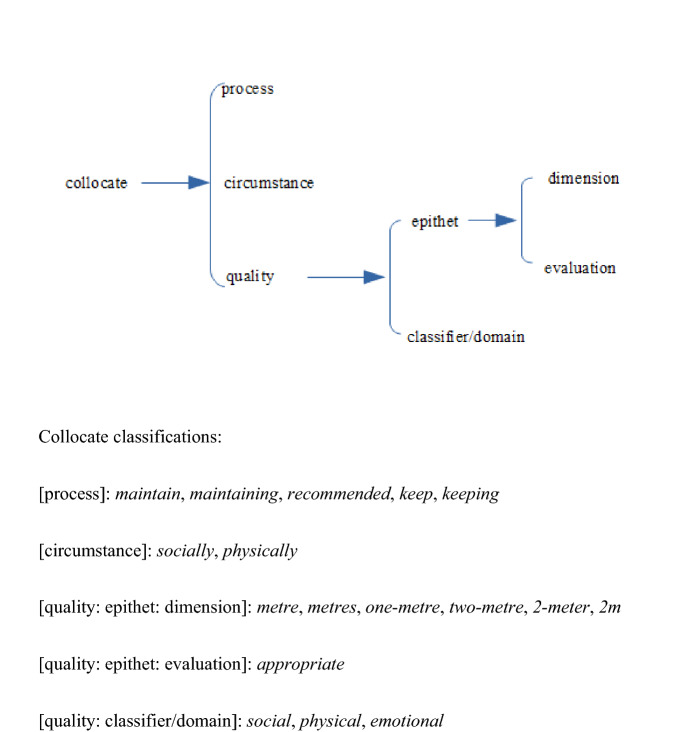
Semantic network of the collocates in the top 40 of NOW-L for *distance* not present in the top 40 of NOW-E

Figure 6 classifes the collocates present in the top 40 for NOW-L but not for NOW-E.

The contrast, not only with NOW-E, but also with BNC and ukWaC50, is clear: while the dimension epithets in the pre-Covid corpora express relative length, there are now additional frequent collocates in NOW-L which express concrete measurements. The evaluation epithet *appropriate*, like *safe*, which does occur in the other corpora, expresses a positive judgement of distance which obeys the norm, as imposed and followed (see the processes expressed by the verbal collocates). The classifier *emotional* makes it to the top 40. Apart from the latter, all collocates precede spatial *distance*. See further Section “Social and Physical Distance/Distancing”.

### Distancing

In assessing the quantitative importance of collocates we will adopt a cut-off frequency of 50 for ukWaC50 and 5 for the other three corpora, in view of the fact that ukWaC50 is an order of magnitude larger than the others.

In BNC, ukWaC50 and NOW-E, where *distancing* does not have a high frequency, the words immediately preceding it are largely grammatical rather than lexical, none of the lexical ones exceeding the cut-off frequency for the corpus. For NOW-L the picture is dramatically different, in line with the huge quantitative importance of *distancing* in this corpus. The most frequent collocates are *social* (F=19,088) and *physical* (751), as shown in Table 1. Other collocates with frequency greater than the cut-off point are: *socially* (119), *safe* (58), *two-metre* (56), *metre* (18), *population* (16), *enact* (14), 2m (14), *strict* (13), *political* (10), *physically* (9), *spatial* (6), *enforce* (6), *appropriate* (6), *current* (6), *maintain* (5). The link with the pandemic is thus very clear. In terms of the network in Fig. 6, which is still appropriate here, we have the following classifications:

[process]: *enact, enforce, maintain*
[circumstance]: *socially, physically*
[quality: epithet: dimensional]: *metre, two-metre, 2m*
[quality: epithet: evaluation]: *appropriate, current, safe, strict*
[quality: classifier: domain]: *social, physical, political, spatial, population *

### Social

#### Frequencies

We again investigate the 40 most frequent lexical collocates, now at the position immediately to the right of *social*.^6^ As with *distance* there are also coordinated sequences, a sub-class of which are discussed in Section “Both Physical and Social Distancing”.

**Fig. 7:**
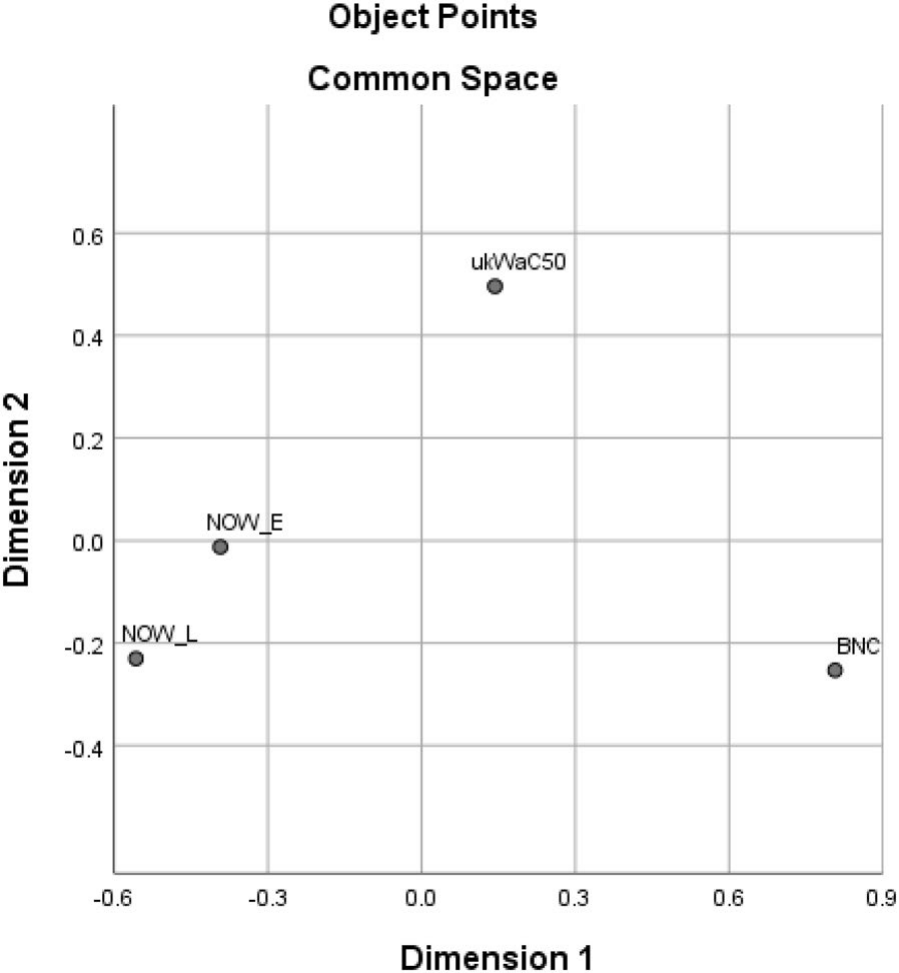
Multidimensional scaling plot of distances between corpora on the basis of numbers of shared collocates in top 40 for *social*

Table 4 shows all the collocates found in the top 40 for any of the four corpora and which top 40 list(s) they occur in, and Table 5 indicates how many collocates are shared by the top 40 of each pair of corpora, while Fig. 7 presents the corresponding multidimensional scaling plot. It can be seen that pairs of corpora share between 45% (BNC/NOW-L) and 80% (NOW-E/NOW-L) of their top 40 collocates.

**Table 4:** Presence of lexical collocates immediately after *social* in top 40 for each corpus

Collocate	BNC	ukWaC50	NOW-E	NOW-L	Collocate	BNC	ukWaC50	NOW-E	NOW-L
Care	×	×	×	×	Networks			×	×
Change	×	×	×	×	Relations	×			
Club	×	×	×	×	Structure	×			
Democratic	×	×	×	×	Order	×			
Development	×	×	×	×	Service	×			
Life	×	×	×	×	Classes	×			
Policy	×	×	×	×	Anthropology	×			
Research	×	×	×	×	Conditions	×			
Science	×	×	×	×	Scientists	×			
Sciences	×	×	×	×	Control	×			
Security	×	×	×	×	Status	×			
Services	×	×	×	×	Affairs	×			
Welfare	×	×	×	×	System	×			
Work	×	×	×	×	Charter	×			
Worker	×	×	×	×	Group	×			
Workers	×	×	×	×	Studies	×			
Enterprise		×	×	×	Exclusion		×		
Events		×	×	×	Inclusion		×		
Housing		×	×	×	Activities		×		
Interaction		×	×	×	Skills		×		
Issues		×	×	×	Capital		×		
Justice		×	×	×	Psychology		×		
Responsibility		×	×	×	Forum		×		
Democrats	×		×	×	Movements		×		
Mobility	×		×	×	Landlords		×		
History	×	×	×		Plugins			×	
Problems	×	×	×		Credit			×	
Behaviour	×	×			Homes			×	
Class	×	×			Commentary			×	
Context	×	×			Democrat			×	
Fund	×	×			Distancing				×
Groups	×	×			Distance				×
Enterprises		×	×		Contact				×
Cohesion			×	×	Gatherings				×
Impact			×	×	Restrictions				×
Isolation			×	×	Protection				×
Media			×	×	Interactions				×
Network			×	×	Insurance				×
Networking			×	×

**Table 5:** Numbers of collocates of *social* shared by top 40 lists for each pair of corpora

	ukWaC50	NOW-E	NOW-L
BNC	23	20	18
ukWaC50		26	23
NOW-E			32

**Fig. 8:**
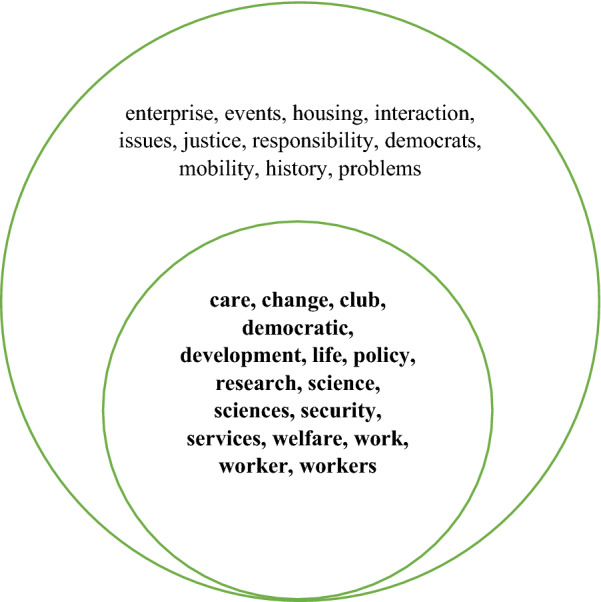
The collocates present in the top 40 for *social* in all four corpora (bold) and those present in three corpora

Table 4 shows that 16 collocates are present in the top 40 of all four corpora. Adding in the collocates which are present in the three corpora gives a further 11. These 27 collocates are shown in Fig. 8. Once again, this suggests that there is a set of items which represents a stable core of collocational usage across the whole time period spanned by our corpora.

**Fig. 9:**
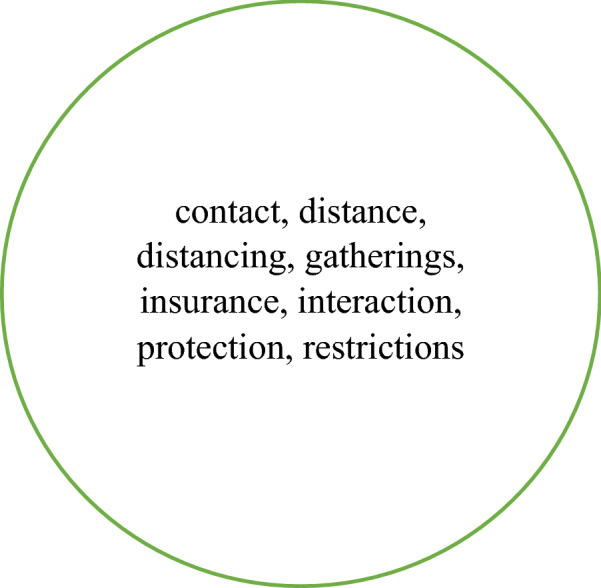
Collocates of *social* which are unique to the top 40 in NOW-L

Table 4 also shows that there are eight collocates which are unique to NOW-L, as shown in Fig. 9.

#### Semantic Analysis

**Fig. 10:**
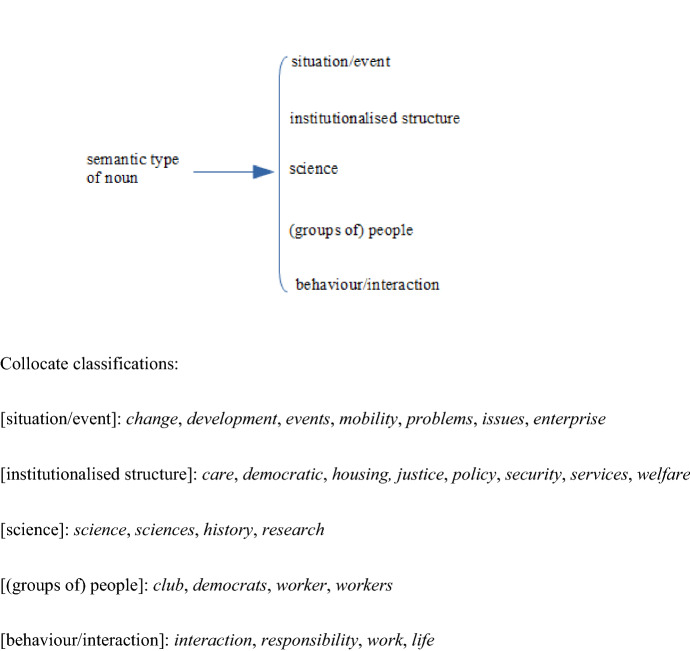
Network for the collocates of *social* occurring in the top 40 in three or all four corpora

Figure 10 gives a semantic classification of the 27 collocates which occur either in all four top 40 lists or three of them.

*Social* is a classifier, which means that it tends to collocate with abstract nouns rather than concrete objects or persons. *Social worker(s)* is no exception in that *social* does not function here as an epithet (* The worker is social. Cf. Tucker (1998: 151) on criterial characteristics of epithets and classifiers) but is a derivation of *social work*. A general definition of *social* is ‘relating to human society’, ‘relating to interactions between members of a society’. The shared collocates show a general pattern of *social* as qualifying the organisation of human society by means of structures, hierarchies, groupings. The top three in BNC are *services*, *security* and *work;* the top three in ukWaC50 are *care*, *services* and *work* and in NOW-E *media*, *care* and *security.* This shows that the institutionalised structures and their functioning are most frequent in these corpora. Furthermore, the collocates *care, justice*, *responsibility*, *services, security, welfare, work* have a clear positive meaning, i.e. the collocations with *social* not simply mean ‘referring to society’ but ‘to the benefit of people’.

Let us now look at the set of collocates which are unique to the top 40 in NOW-L, shown in Fig. 9. *Contact, distance, distancing, gatherings, interaction, restrictions* can be classified as related to the behaviour of, and interaction between, members of a society. *Insurance* and *protection* belong to institutionalised structures, the mechanisms to deal with social issues.

We checked which of these collocates (apart from *distance* and *distancing*) are connected with the pandemic. All instances of *social restrictions* in a 100 sample are pandemic-related; in the sample of 100 *social gatherings* there were 6 cases not pandemic-related. The 94 remaining ones all occur in the context of restrictions; in a sample of 200 *social contact* instances there are only three non-pandemic related ones**;** *social insurance* occurs in both pandemic-related and -unrelated contexts, and its frequency is clearly heightened because of new insurance regulations; apart from just a few instances, all *social interaction* cases in a 200 sample were Covid-related; in a 200 sample of instances of *social protection* the majority are Covid-related, in the context of new social protection schemes. In conclusion, all of the eight collocates unique to NOW-L make it to the top 40 as a result of the focus on Covid-19.

The collocation *social responsibility* (in the top 40 of all but BNC ) occurs in a wider variety of contexts, but it is safe to conclude from examination of a concordance that the Covid-19 crisis has contributed to its frequent appearance in NOW-L. *Social workers* likewise frequently crops up in Covid-19 contexts, mainly referring to frontline staff. What we see then is the emergence of ‘new’ collocations (in the sense of their being absent in the top 40 in other corpora) on the one hand, and an increase of ‘established’ collocations on the other. The ‘new’ ones all have to do with government measures to combat the spread of the virus.

### Physical

#### Frequencies

We will once more proceed to look at the 40 most frequent lexical collocates, now at the position immediately to the right of *physical*. We again defer a subset of coordinated sequences for later discussion (Section “Both Physical and Social Distancing”).

**Fig. 11:**
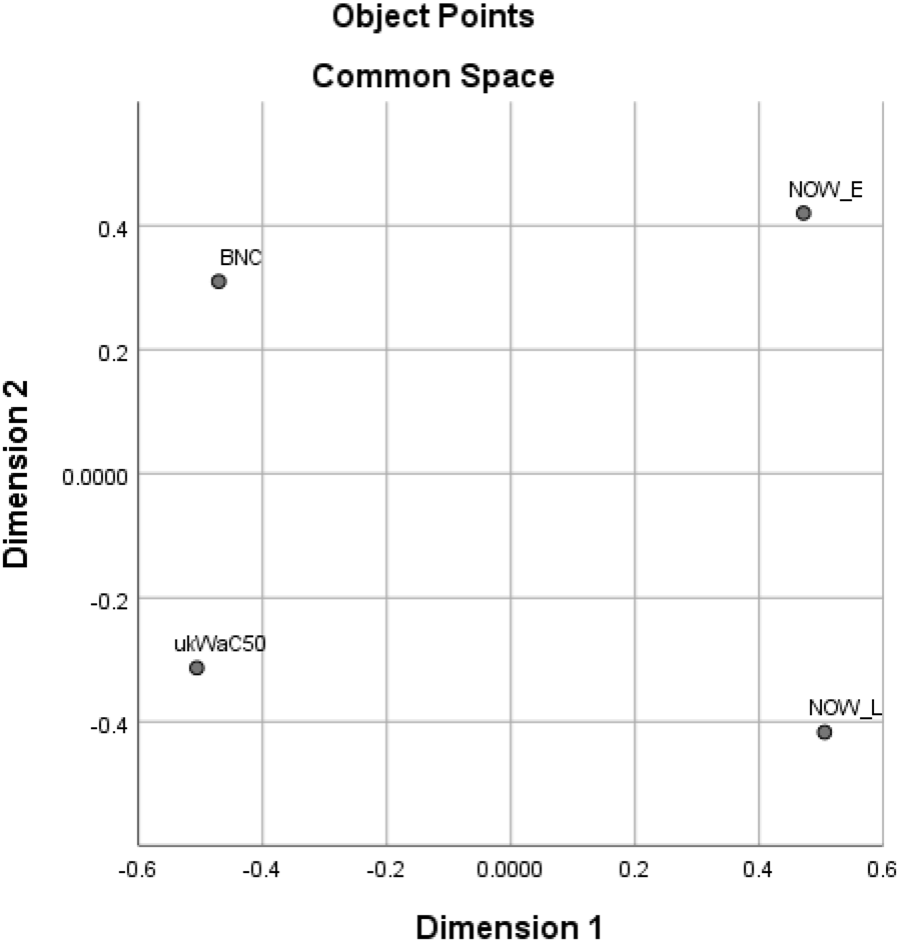
Multidimensional scaling plot of distances between corpora on the basis of numbers of shared collocates in top 40 for *physical*

Table 6 shows all the collocates found in the top 40 for any of the four corpora and which top 40 list(s) they occur in, Table 7 indicates how many collocates are shared by the top 40 of each pair of corpora, and Fig. 11 presents the corresponding multidimensional scaling plot showing how close or distant the pairs of corpora are with respect to the number of collocates shared by that pair.

**Table 6:** Presence of lexical collocates immediately after *physical* in top 40 for each corpus

Collocate	BNC	ukWaC50	NOW-E	NOW-L	Collocate	BNC	ukWaC50	NOW-E	NOW-L
Abuse	×	×	×	×	Stores		×		×
Activity	×	×	×	×	Training		×		×
Appearance	×	×	×	×	Copy			×	×
Condition	×	×	×	×	Evidence			×	×
Contact	×	×	×	×	Gold			×	×
Damage	×	×	×	×	Wellbeing			×	×
Disabilities	×	×	×	×	Geographers	×			
Disability	×	×	×	×	Conditions	×			
Education	×	×	×	×	Needs	×			
Exercise	×	×	×	×	Reality	×			
Fitness	×	×	×	×	Word	×			
Health	×	×	×	×	Recreation	×			
Injury	×	×	×	×	Theatre		×		
Presence	×	×	×	×	Processes		×		
Violence	×	×	×	×	Therapy		×		
Access		×	×	×	Review		×		
Space		×	×	×	Laboratory		×		
Attributes	×		×	×	Development		×		
Harm	×		×	×	Infrastructure			×	
Strength	×		×	×	Attacks			×	
Symptoms	×	×		×	Injuries			×	
Problems	×	×	×		Change			×	
World	×	×	×		Game			×	
Body	×	×			Assault			×	
Characteristics	×	×			Release			×	
Chemistry	×	×			Performance			×	
Environment	×	×			Comedy			×	
Examination	×	×			Media			×	
Features	×	×			Distancing				×
Geography	×	×			Distance				×
Properties	×	×			Loss				×
Science	×	×			Copies				×
Sciences	x	×			Side				×
Form	×		×		Meeting				×
Pain	×		×		Proximity				×
Punishment	×		×		Barriers				×
Activities		×		×	Proof				×
Location		×		×	Meetings				×
Security		×		×

**Table 7:** Numbers of collocates of *physical* shared by top 40 lists for each pair of corpora

	ukWaC50	NOW-E	NOW-L
BNC	28	23	19
ukWaC50		19	22
NOW-E			25

**Fig. 12:**
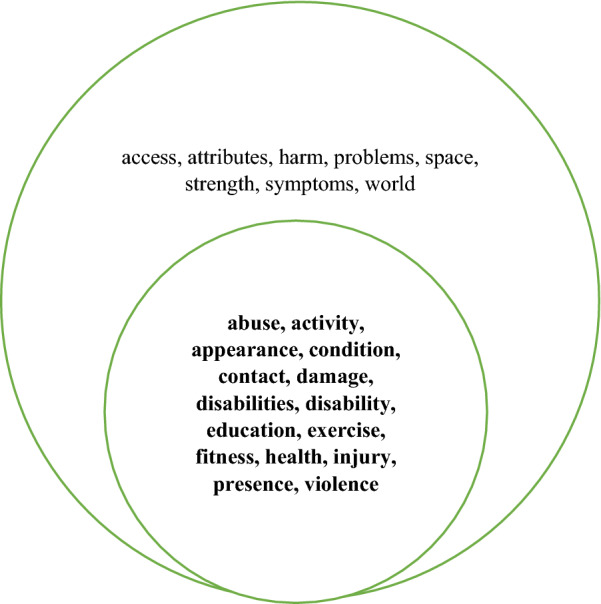
The collocates of *physical* present in the top 40 for all four corpora (bold) and those present in three corpora

**Fig. 13:**
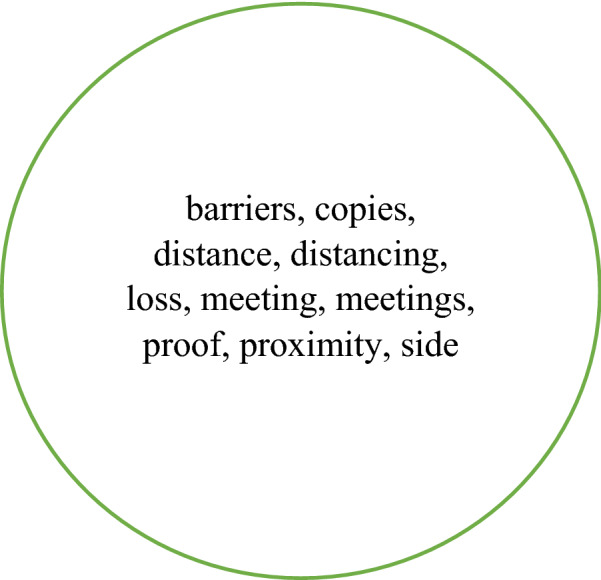
Collocates of *physical* which are unique to the top 40 in NOW-L

As shown in Table 6, 15 collocates are common to all four corpora and eight appear in three of them. These are presented in Fig. 12. The 10 collocates which are unique to NOW-L are shown in Fig. 13.

#### Semantic Analysis

**Fig. 14:**
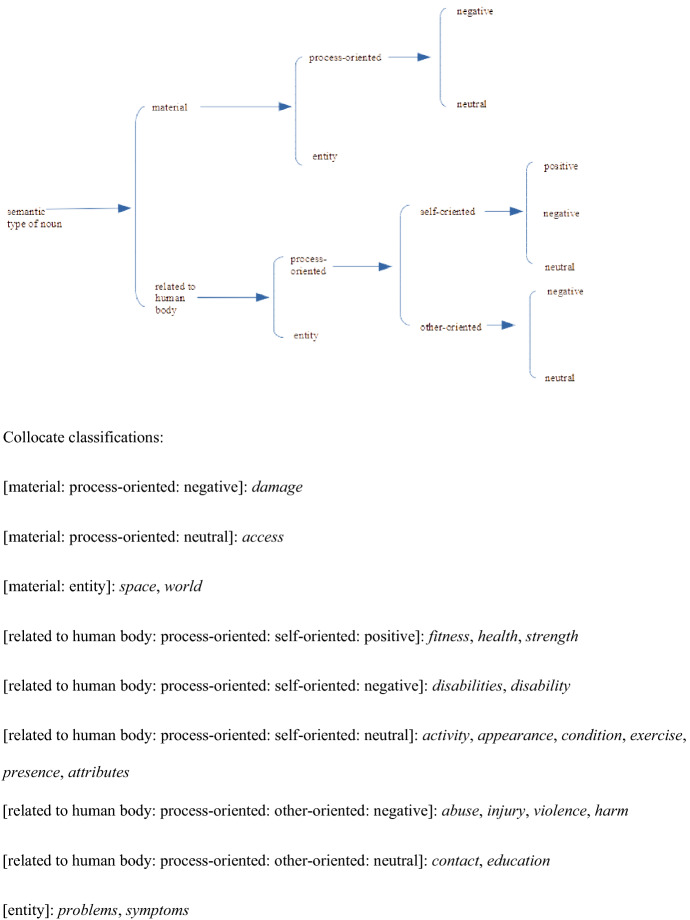
Network for the collocates of *physical* occurring in the top 40 in three or all four corpora

Figure 14 shows a semantic network for the lexical collocates immediately following *physical* which occur in three or all four corpora. The large majority of the collocates shared by three or even all four of the corpora express processes related to the human body. These processes are either relational (*to be fit, healthy, strong*, etc.) or material (*to exercise, to abuse (someone),* etc.). Out of the 23 collocates, 9 have a negative meaning: *damage, disabilities, disability, abuse, injury, violence, harm, problems, symptoms.*

In our key terms *physical distance* and *physical distancing, physical* has the meaning ‘related to the human body’ and *distance* is a Thing, while *distancing* is a nominalised process which is in between self- and other-oriented: ‘somebody distances themselves from somebody/something’. These key terms are discussed in Section “Physical Distance/Distancing”.

We checked which of the eight collocates (i.e. apart from *distance* and *distancing*) which are unique to NOW-L are related to Covid-19. *Physical barriers* is connected with the pandemic in three out of four occurrences; all instances of *physical meeting* are Covid-19 related. The collocation contrasts with *virtual meeting*; all instances of *physical meetings* are likewise pandemic-related, in contexts of prohibitions and limitations; *physical proximity* is Covid-19 related in three out of four occurrences; *physical copies* appears in the top 40 of NOW-L as a result of concern about the limited availability of physical copies (in contrast with digital ones) especially of games; *physical loss* is a term in insurance regulations and appears in NOW-L especially in the context of court rulings regarding claims of physical loss due to business interruptions and virus contamination connected with Covid-19. Of the 10 collocates unique to NOW-L only *physical proof* and *physical side* are not demonstrably Covid-19 connected. *Barriers, copies* are material entities; *loss, meeting, meetings, proximity* are process-oriented. All six collocates are used in contexts of restrictions on interaction.

### Social and Physical Distance/Distancing

#### Social Distance/Distancing

The preceding discussion has been concerned with the properties of the separate key items. In this section we zoom in on the collocations *social distance/distancing*.

The BNC has 38 occurrences of *social distance,* and *social* ends up in position 10 of the most frequent collocates of *distance*; ukWaC50 has 105 instances, but *social* comes in the 35^th^ position only as a collocate of *distance* in this corpus; NOW-E has only one instance. As to the usage of *social distance* in BNC, we pointed out above (Fig. 3) that it has both a spatial and a non-spatial meaning in the data. The frequencies of either usage, however, reveal the prototypical meaning of *social distance* in this corpus as non-spatial: out of the 38 instances only one is spatial. This is the passage, which comes from a lecture in the social sciences:

(7) Yeah, gesture, right. What else ? You must have thought of some more than that. What about issues about personal space ? **Social distance**, yeah? Okay, you'll find there're big er cultural differences there. Okay, we're we're a non, in the UK as a culture we don't stand very close to people but actually women stand a lot closer to women than women to men or men to men. Erm, touch? (BNC JT0 347)

The non-spatial occurrences refer to separation between socio-economic class (example 8) or to closeness to ingroup vs. non-acceptance of outgroup (example 9). The meaning differs slightly depending on whether the focus is more on economic factors or on cultural ones, and more on facts or on attitudes, but the overall sociological sense of ‘the extent to which individuals or groups are removed from or excluded from participating in one another's lives’ (https://www.dictionary.com/browse/social-distance) covers the uses encountered. The senses in which the collocation is used can be found in one or another of the technical definitions given to the term in sociology (https://www.sociologyguide.com/basic-concepts/Social-Distance.php) or social psychology (https://www.thoughtco.com/social-distance-3026589).

(8) This ‘rap gap’ reflects a growing divide within the African-American community. The income difference and **social distance** between middle class blacks and those classified as poor is now larger than ever before in post-slavery history. (BNC ACN 1818)
(9) Social psychologists have conducted thousands of studies about stereotypes of outgroups or desired **social distance** from outgroup members, etc . They have built up an enormous collection of data about the images which subjects, especially white male Americans, have about outgroups. (BNC FA9 1312)

In ukWaC50 the same pattern is found: the large majority of instances of *social distance* are non-spatial. The following examples make this non-spatial interpretation explicit:

(10) Cities entail **social distance** between thousands of physically proximate individuals. (text 2321640)
(11) In fact, Peach (1975, p1) contemporaneously based his book of classic segregation readings on the similar hypothesis that spatial segregation is positively correlated with **social distance**–and therefore negatively correlated with assimilation (text 2331756)

Out of the 105 occurrences seven are spatial. Here are two examples:

(12) **social distance**–1.25–4 m the distance for casual interactions, business dealings, shopping and so on. Verbal cues still available but many of the others are lost. People will avoid sitting next to others on buses because it may be seen as an invasion of this space. (text 1268815)
(13) **social Distance**. 1.22 to 3.66 m. Impersonal, businesslike contacts. Less detailed visual communication. Normal voice level. Touch not possible. (text 285446)

In contrast with example (7) from the spoken BNC above, where *social distance* refers to a scale of physical proximity on which people in different cultures interact, the collocation is used in (12) and (13) with a very specific dimensional meaning. *Social* contrasts here with other types of physical distance: intimate, personal and public. These four scales of distances were defined in Hall (1966) as referring to very specific, measurable physical distances between people. The term *social distance* thus has a technical sense in proxemics.

**Fig. 15:**
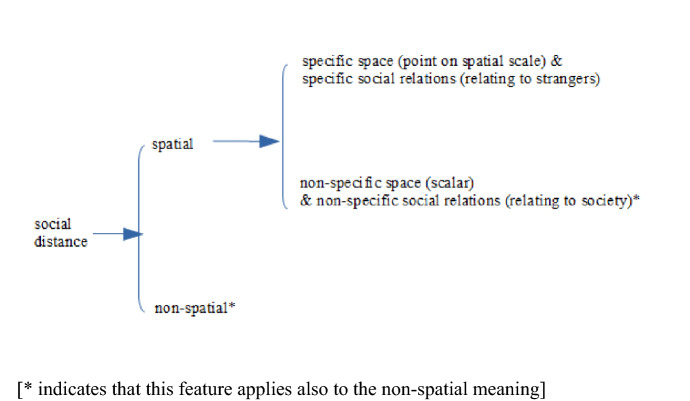
Meanings of *social distance* in BNC and ukWaC50

This technical use adds a sense to the classifier *social* which refines its semantic profile. Its meaning of ‘relating to society’ or ‘relating to interactions between people’ (cf. Section “Semantic Analysis” above) is the one we find in the non-spatial uses of *social distance.* In the spatial uses, however, *social* has two meanings, a general one (‘relating to society’) and a specific one (‘relating to interaction between strangers or new acquaintances’, Hall, 1966). This entails that the collocation *social distance* has different meanings as set out in Fig. 15.

Let us now compare NOW-E and NOW-L. In NOW-E *social distance* occurs only once, and it is an instance of non-spatial distance. It refers to lack of rapport, the absence of a common outlook in different groups in society:

(14) The continuous unsuccessful political debates and increasing emotional polarisation that have followed have further made the **social distance** more apparent. Yet, the in-depth examination of the emotions, that still persist, narrows the distance between generations, and apprehension and uncertainty are the two emotions that are shared by all British citizens. (NOW 19-10-03 blogs.lse.ac.uk)

From the 676 occurrences of *social distance* in NOW-L we have taken a random sample of 200, which allowed us to examine the wider co-text (collocation further to the left and right than just one word in each direction). All 200 instances refer to spatial distance of the specific type with regard to spatiality (see Fig. 15 above). The meaning of *social* is not specified as ‘between strangers’, i.e. it does not have the technical sense with which it is a member of a closed paradigm of four (contrasting with *intimate*, *personal* and *public*), since it includes any person in specific contexts, except people in one’s bubble (intimates). The paradigm has shifted from four to two members: social and intimate.

This meaning defines both the nominal and verbal forms. Within the sample there are 62 instances (31%) of *social distance* as a verb. This usage shows the rapid conversion which has taken place. The grammar shows variation: *to social distance* is mostly used intransitively, but also reflexively (*to social distance oneself*), and (rarely) transitively with an implicit goal (*to social distance (people)),* as in the following:^7^

(15) workplaces **have to social distance** and buses do, and supermarkets do–yet schools don't–(NOW 20-05-03 tes.com)

As a noun phrase (69% of the 200 instances) *social distance* typically occurs with processes such as *maintain* (39 occurrences) and *keep* (23 occurrences). The nominal group may include other modifiers, such as *the standard 2 metres recommended social distance, an appropriate social distance, a safe social distance.* In verbal uses the process may be enhanced by circumstantial elements, as in *to appropriately social distance.* The premodifiers and circumstances are in the semantic domains of obligation and norms. Expressions of obligation (*be forced to, required to, need to, have to, should, advise s.o. to, remind s.o. to, encourage s.o. to*, etc.) or possibility (*allow to, can(not), it is (not) possible to,* etc.) are also frequently associated with *social distance* in its verbal use, as in (15) and (16) or, more often, in constructions involving the processes mentioned above, as in (17), (18) and (19):

(16) Those who live alone and are not worried about their own health **should** still **social distance** to prevent spreading the disease to others who may suffer more severely from the virus (NOW 20-03-28 express.co.uk)
(17) Smith-Jarvis has, like many others, been **forced to** work from home and **social distance** during the coronavirus outbreak. (NOW 20-03-23 eadt.co.uk)
(18) **It is required** that the personnel working on the site maintain an appropriate **social distance**, including for purposes of elevators/meals/entry and exits. (NOW 20-04-10 lexology.com)
(19) **It is impossible** to **social distance** with cyclists weaving around pedestrians. (NOW 20-06-18 gov.uk)

In some examples the meaning of *social distance* is clearly ‘physical distance’ (as in (18) and (19) above, while in other examples (such as (16) and (17)), it combines the senses of ‘physical distance’ and ‘fewer contacts outside one’s intimate bubble’. In the latter sense, too, *distance* is purely spatial.

No matches were found for *social distancing* in NOW-E. The contrast with the 19,088 hits in NOW-L is overwhelming. Again, we have looked closely at a random sample of 200 instances. As a process *social distancing* occurs with collocates from the same domains as *to distance*: obligation and possibility. Obligation is looked at from the point of view of the authority (*impose, guidelines, ensure, measures,* etc.) and of those on whom the obligation is imposed (*adhere to, maintain, observe, practise, break,* etc.).

Let us turn to the collocations with the adverb *socially.* Of these, the most frequent one is *socially distanced.* It is used exclusively in Covid-19 contexts. It occurs predicatively as the complement of verbs such as *be* and *remain*, but it is its occurrence in attributive function referring to people, events, as well as locations which shows the extent to which the concept is integrated in the lexicon and is understood by language users. It is doubtful that in pre-Covid-19 times accurate comprehension of such instances as (20) and (21) could have been taken for granted.

(20) Under The Edge have been providing Wotton and the surrounding villages with bouquets via **socially distanced** delivery on Thursdays. (NOW 20-05-19 gloucestershirelive.co.uk)
(21) Cricket is perhaps our most **socially distanced** team sport. (NOW 20-06-23 countypress.co.uk)

*Socially distance* is interesting because *to social distance* also occurs. On the basis of the frequency of verbal usages in the 200 sample, the total frequency of verbal *social distance* can be estimated at 259, which is less frequent than *socially distance* (422). *Socially distancing* is mostly used as a progressive but equally before head nouns such as *measures*, *guidelines*. In those cases it is synonymous with *social distancing*.

#### Physical Distance/Distancing

Table 1 shows that *physical distance* follows the frequency pattern of *social distance:* it is rare until NOW-L. The main differences are, however, that *physical distance* is even rarer in BNC than *social distance*, and that it is comparatively infrequent in NOW-L as compared with *social distance*. In contrast with *social distance*, only a spatial interpretation obviously obtains, so the only question is whether the contexts in which the term is used differ in the pre- and post-Covid-19 data. Let us first look at the 11 occurrences in BNC. In all instances, the collocation is used for contrast–implicit or (in most cases) explicit–with non-physical distance. Since unmodified *distance* is prototypically spatial in that corpus (see Section “Semantic Analysis” above), the specification *physical* occurs when a potential non-spatial interpretation which might arise from the context is emphatically rejected. All 11 instances are found in academic or non-academic social science writings. As it is precisely in those same genres that *social distance* is used in a non-spatial sense, this explicit modification is unsurprising. The following examples illustrate the usage of the collocation in BNC.

(22) Their lives would take them far apart from each other and from Curry Rivel itself—yet they would remain a **close-knit family**, for all the **physical distance** which separated them. (BNC CBJ 1090)
(23) On the contrary, one sees how greater **physical distance** could well help to bring **emotional relationships** closer. (BNC AP7 444)

The way in which *physical distance* (92 instances) is used in NOW-L is totally different. It is used in the same way and with the same meaning as *social distance* in its spatial sense. Nearly all occurrences are Covid-19 related and in the immediate co-text words from the field of obligation are frequent: *measures* (10 occurrences), forms of the lemma *maintain* (32), forms of *keep* (12). Also specific measurement items are frequent: *metre(s)* (in combination with *one*/*two*/*2*) (11), *feet*/*foot* (preceded by *six*/*6*) (11). No apparent semantic difference with *social distance* emerges. The only noteworthy pragmatic usage point–though not in frequency terms–is the occurrence of *physical distance* in a context of contrast with *psychological distance*. Although there are only three such cases in NOW-L, there are none at all in the random sample of 200 of *social distance*, and it is unlikely there would be, since *social distance*–unlike *physical distance*–is potentially non-spatial. Here is an example:

(24) The first obstacle I identified was that **physical distance** leads to **psychological distance**. This is a stylised fact already well established in the behavioural science literature. (NOW 20-05-05 blogs.lse.ac.uk)

Hence, while in the Covid-19 context *social distance* and *physical distance* are–at least in many cases–synonymous, their respective total semantic make-up leaves the possibility of differential behaviour when the need arises. It also leaves the possibility of individual preferences of language users for one term or the other. A grammatical difference between the forms *social distance* and *physical distance* is that the former but not the latter not only occurs as a noun but also as a verb. *Distance* as a verb only selects *physically*.

Table 1 shows that the pattern for *physical distancing* is again similar to that for *social distancing* in that it is absent (with one single exception in ukWaC50) from the pre-corona corpora, and suddenly appears in NOW-L. Its much lower frequency in that corpus as compared with *social distancing* is, however, noticeable. Equally noticeable is the higher frequency of *physical distancing* in comparison with *physical distance.* Again, this is parallel with the difference between *social distance* and *social distancing.*

There are 751 occurrences of *physical distancing* and 9 of *physically distancing* in NOW-L (see Table 1). This points to a nominal versus a verbal use of *distancing*. Let us first look at the 9 cases of *physically distancing.* Its occurrence is exclusively Covid-19 related. In some cases the collocation expresses a process in progress, notably when it occurs after the conjunction *while/whilst* (6 cases), as in example (25). It also follows prepositions, as in (26):

(25) I’m so for it, let people eat and drink outside while **physically distancing**. (NOW 20-06-13 telegraph.co.uk)
(26) That means accepting responsibility for protecting yourself, your family and your community by **physically distancing** in the community, cleaning your hands, covering coughs and sneezes and staying at home and away from others when you feel sick (NOW 20-05-11 hulldailymail.co.uk)

The verbal form also occurs in the collocations *physically distance* (16 instances) and *physically distanced* (22 instances). This shows that as a verbal collocation it has become entrenched in the language.

(27) it is vitally important that people can **physically distance** for those essential trips or for exercise (NOW 20-05-13 thecourier.co.uk)

*Physically distanced* applies to people, places and activities in the same way *socially distanced* does. The following example testifies to the creative use of what has become a familiar expression whose understanding can be taken for granted:

(28) Has an unconscious measure of performance (a key source of behaviour!) been how many meetings you can schedule in a day–is that staying the same in your now **physically distanced** world, or might this be a chance to change the system? (NOW 20-04-08 blogs.lse.ac.uk)

For a closer examination of *physical distancing* a random sample of 200 instances was taken. No difference was found between the usage of this collocation and that of *social distancing.* All instances are Covid-19 related and occur in the context of obligation, with such items as *measures* (40 instances), *rules* (11), *guidelines* (8), *requirements* (5), *safe* (18), *appropriate* (7) and forms of *maintain* (32), *practice* (5) and *observe* (6)*,* in the immediate co-text. As with *social distancing*, concrete measurement items may accompany the collocation, in particular *metre*(*s*) (16 instances).

#### Both Physical and Social Distancing

In this section we look at examples where *social*, *physical* and *distance/distancing* all co-occur.

The BNC has only one occurrence, which is the following:

(29) The tragedy of these kinds of development is that the opportunity may be lost of creating an integrated community, and of benefiting from the social advantages that can accrue from good design (Masser and Stroud 1965). It is not the designs that are important but their social impact; and it is not the **physical distance** between new and old that is of concern, but the **social distance** (BNC FB2)

There is a clear contrast between *physical distance* and *social distance*, the former referring to spatial, the latter to non-spatial distance, i.e. the contrast between a separation in space and a cultural-psychological divide. This is the semantic difference which we find in all 18 instances in ukWaC50 as well. Either this difference is expressed in the context of an opposition or in the context of a causal relation: either “physical but not social” or “physical and hence social”. This shows that physical distance is often seen as causing social distance or estrangement.

In NOW-E, we surprisingly did not encounter one single instance. There are 55 examples where *physical* and *social* co-occur within 5 words of each other, but they are not collocates of *distance* or *distancing.* The semantic difference between the two items is, however, the same and just as clear as in BNC and ukWaC50. Examples (30) and (31) illustrate the co-occurrence of the items in this corpus:

(30) Michael J Halloran writes that the intergenerational cultural trauma caused by 300 years of slavery–alongside poor economic circumstances and social prejudice–has led to the poor state of **physical, psychological and social** health among African Americans. (NOW 19-12-28 blogs.lse.ac.uk)
(31) Our city has undergone tremendous **physical and social** transformation across the past two decades. (NOW 19-11-28 eveningtimes.co.uk)

The situation changes radically in NOW-L. There we find 63 instances of the two items in close proximity of *distancing* and 5 in the proximity of *distance*. But more interesting than the mere frequency is the change in the semantics. Whereas in the other corpora the distinction between *physical* and *social* was evident, this is no longer the case in NOW-L. In some cases the terms seem to refer to exactly the same situation/phenomenon, in others one must guess at a possible nuance which is never made explicit. In other words, the terms *social distance/distancing* and *physical distance/distancing* are used in coordination with *and* as well as with *or*, without a context which would clarify the difference between them, or indeed whether *or* means disjunction or synonymy. In example (32) the coordinating *and* implies a difference between the terms, but it is clearly not the one encountered in the other corpora. In both cases *distancing* is spatial.

(32) “Contact tracing is key especially as the UK starts to relax the **social and physical distancing** measures,” Dr Kluge told The Guardian. (NOW 20-06-15 walesonline.co.uk)

Nevertheless the difference is not clarified, and in other contexts the terms appear to be synonymous. Compare:

(33) Businesses that involve mass gatherings and **physical contact** where **social distancing** would be difficult to control will not be permitted to resume operations. (NOW 20-06-17 lexology.com)

It is possible that (33) uses social distancing to mean ‘reduction in the number of people present’ rather than ‘reduction of the space between people’, but this is not made clear.

Synonymy is also apparent in *or* coordination, as in (34):

(34) **Social or physical distancing** has affected billions of people around the world during the past weeks. The Government has told us to stay at least two metres away from each other, “to stop the spread” of the coronavirus. (NOW 20-05-17 churchtimes.co.uk)

The two terms are in some cases juxtaposed as modifiers of *distancing* without a linking conjunction, as in (35)

(35) Amidst **physical social distancing**, non-physical socialising has flourished (NOW 20-05-19 edp24.co.uk)

In (35) above, the implication is that there is physical and non-physical social distancing as well as physical and non-physical social contact. Nevertheless, *social distancing* is still used in a spatial sense, in our data unique to this corpus, but the addition *physical* indicates that the writer felt it necessary to make that interpretation explicit.

In conclusion, NOW-L behaves very differently from the earlier data both in terms of the emergence and frequency of *physical* and *social distance/distancing* in each other’s proximity, and through the lack of clear boundaries between the terms. The fluidity between the concepts is reflected in the grammatical as well as the pragmatic patterns and contexts in which they occur together^8^.

## Concluding Remarks

Our analyses have shown that three of our key items, *distance*, *social* and *physical*, each have a set of collocates at the appropriate positions which is stable across the three decades spanned by our four corpora and can therefore be regarded as constituting the core collocational behaviour of these three word forms, at those positions, in British English. For each word, these collocates fall into a small number of semantic classes, as detailed in Sections “Semantic Analysis”, “Semantic Analysis”, “Semantic Analysis”.

The availability of part of the NOW corpus containing material from the .uk domain for the first six months of 2020 allowed us to study any changes occurring in the behaviour of our key terms in this period and to use concordances to find out whether such changes were related to the Covid-19 pandemic. Particularly useful were comparisons of NOW-L with NOW-E, taken from the last six months of 2019, and especially the analysis of collocates that made it to the top 40 only in NOW-L.

For the key items *distance*, *social* and *physical*, the great majority of the collocates unique to the top 40 of NOW-L have been shown to be directly relatable to the pandemic.

*Social distance* is rare in the pre-Covid corpora (range 0.01–0.34 wpm). Its prototypical use is non-spatial and is concerned with social relations. It is, however, much more frequent in NOW-L (4.41 wpm), its use now being spatial and concerned with the opposition between a distance required by the pandemic restrictions and smaller, intimate distance.

*Physical distance* is rarer than *social distance* even in NOW-L, where the two expressions are often used interchangeably.

*Distancing* is rare in all but NOW-L, where it is very frequent and collocates most frequently with *social* and *physical*, other collocates also being Covid-related.

Examples in NOW-L where both *social* and *physical* co-occur with *distancing* reveal that in some cases *social distancing* and *physical distancing* are being used as synonyms, while in other cases the relationship is left unclear.

On a more general level, an important conclusion from our work is that the semantic fields of our key items have witnessed clear shifts in the post-Covid period in terms of synonymy, antonymy and associated concepts. At the same time the grammar of some of the items has undergone shifts.

In future work we hope to expand on the observations made in the present article. Possible avenues for further exploration include the use of our key terms in scientific publications and the extension of our studies to other European languages. Usage in scientific–especially medical–publications should shed light on the development of the terms *social/physical distancing* from their source domain into everyday discourse. Further work on the theoretical implications of the type of change described in this paper is also called for. Several studies in ‘distributional semantics’ (e.g. Gries & Divjak, 2009; McDonald & Ramscar, 2001) have shown that semantic change can be studied by charting collocational networks. However, changes described have typically been gradual. The sudden and radical nature of the changes reported here is a challenge for further elaboration and discussion within the framework of distributional semantics^9^.

## Data Availability

All data used were from freely available corpora.

## Footnotes

1 We are not interested here in the mutual attraction between our search items and their collocates, but only in how often particular words appear in the relevant position just before or after the search item. The software provided for analysis of the corpora does not include any directional measures of collocational strength such as delta-P. We therefore use the simple frequencies of collocates.

2 The decision to examine the top 40 collocates rather than, say, the top 10 or 100, was taken in order to achieve a balance between coverage and a manageable quantity of data.

3 Multidimensional scaling (MDS) is “a technique that is designed to construct a ‘map’ showing the relationships between a number of objects, given only a table of distances between them” (Manly, 1994: 168; for further detail and examples of use in linguistic work see e.g. Butler & Gonzálvez-García, 2014, Chapter 4). In our case the distances are calculated by the program from the matrix of the number of collocates each pair of corpora share. It is clear from Figure 1 that the most similar pair are ukWaC/NOW-E, while the least similar are BNC/NOW-L.

4 We present our semantic analyses in the form of networks in order to show oppositions between different semantic types of collocates rather than simply tagging them semantically, for example with the Wmatrix software, so leaving the oppositions implicit.

5 Networks which are for non-adjectival items such as *distance* clearly cannot be based on categories from Tucker’s work, since this was confined to adjectives. Rather, they reflect our own classifications of the items concerned.

6 In view of the large size of ukWaC50 and the high frequency of *social* in it (378,955) we took a random selection of 100,000 lines from the concordance for *social* as a basis for the analysis.

7 This example is not in the sample of 200 taken for detailed analysis, but is included here to emphasise the variety of constructions which are now well understood.

8 Our work-in-progress on the terms in research reports will throw light on whether a clearer distinction is made in scientific publications.

9 We are grateful to the anonymous reviewer who has drawn our attention to the potential interest of our corpus results for further work on the theoretical implications.
